# The contribution of interprofessional education in developing competent undergraduate nursing students: integrative literature review

**DOI:** 10.1186/s12912-023-01482-8

**Published:** 2023-09-14

**Authors:** Nombulelo Esme Zenani, Leepile Alfred Sehularo, Gopolang Gause, Precious Chibuike Chukwuere

**Affiliations:** 1 NuMIQ Research Focus Area, School of Nursing Science, Faculty of Health Sciences, North-West University, Mmabatho, North West Province South Africa; 2 NuMIQ Research Focus Area, School of Nursing Science, Faculty of Health Sciences, North-West University, Potchefstroom, North West Province South Africa

**Keywords:** Competence, Contribution, Developing, Interprofessional education, Nursing education, Undergraduate nursing students

## Abstract

**Background:**

Effective interprofessional team collaboration is one of the necessary domains for successful interprofessional collaborative practices in healthcare (IPCP), which is crucial for the delivery of safe and quality healthcare services. Therefore, understanding the contribution of interprofessional education in nursing students is vital to improving collaboration practices in nursing students, in preparation for the dynamics that await after registration in practice amongst the interprofessional team. Thus, the aim of the study was to summarise the contribution of interprofessional education in nursing education in developing competent undergraduate nursing students.

**Design:**

The integrative literature review design as described by Whittemore and Knafl was adopted for the review. The review consists of five steps, namely, problem identification, literature search, data analysis, data interpretation, and the last step was data presentation. Three databases were searched for the articles, namely CINHAL, Scopus, and Science Direct. Articles were retrieved using Search terms such as “Competence,” “Contribution,”, “Development,” “Interprofessional education” and “Undergraduate nursing students” retrieved Articles published between 2018 and 2022 were selected.

**Results:**

Three themes emerged from the review, namely the promotion of patient safety in nursing practice, the socialisation of nursing students in interprofessional collaboration, and the promotion of the development of professional identity.

**Conclusion:**

This study is the first step in determining the contributions of early interprofessional education to nursing education. It could set the stage for further studies that examine strategies the undergraduate nursing curriculum can adopt and develop sound interprofessional competencies that promote patient safety and quality healthcare by nursing students.

**Impact:**

The developers of the nursing curriculum and nursing educators can use the results in developing a curriculum that includes interprofessional education with the aim of improving the quality of teaching and learning that advances competent and safe nursing students.

## Introduction

The ever-evolving healthcare system including mental health care, with the increasingly complex health needs of patients and communities, requires a great deal of innovative and effective concepts of patient care. Effective patient care across all healthcare systems such as in mental health nursing is crucial in facilitating positive nurse and patient outcomes. Effective patient care concepts require key competencies, such as effective communication, teamwork, and interprofessional collaboration between healthcare professionals [1]. Thus, improving interprofessional collaboration with the aim of strengthening effective interprofessional clinical practice (IPCP) in healthcare is one of the approaches that can mitigate challenges related to complexities related to delivering quality medical care for all healthcare users such as mental health care users [2]. The World Health Organization (WHO) defines IPCP as a practice in which multiple healthcare professionals work with each other, share goals, have clear roles and responsibilities, and demonstrate effective teamwork, communication, and shared decision-making [3]. In nursing education, IPCP settings are clinical environments in which nursing students with other healthcare professionals work together in a coordinated manner, and develop, execute, and evaluate continuously comprehensive patient-centered care plans with each other [4]. Interprofessional collaboration is key in avoiding fragmented care that opens a gap for potential adverse events in the care of patients and communities, thus regarded as a key to improving healthcare [1]. The implementation of IPCP can be challenging due to insufficient healthcare professionals, non-coordinated and segregated curricula of the various healthcare professionals, and insufficient resources to teach and implement IPCP [4], [1].

The nursing students form part of the IPCP team within the clinical areas and developing the IPC competencies of nursing students contributes to providing safe and quality healthcare whilst working in an interprofessional collaboration team. Thus, the aim of this study was to summarise global literature to synthesize the contribution of interprofessional education in nursing education in developing competent undergraduate nursing students.

## Background

It is imperative for undergraduate nursing students to be socialized in working competently in interprofessional teams, where they can exhibit collaborative practices that promote patient safety, and optimal patient outcomes and thus strengthen health systems [5]. Furthermore, the literature reveals that interprofessional collaboration practices help to decrease fragmented communication, promote teamwork, and enhance quality improvement practices between the nursing students with the rest of the interprofessional team [5], [6], [7]. Interprofessional collaboration (IPC) is a partnership between professionals from diverse backgrounds who possess distinctive professional cultures, who work together to solve problems or provide services [8]. The World Health Organization (WHO) suggests that IPC occurs when a variety of healthcare providers work together with families and communities to provide comprehensive healthcare services and the highest quality of care across all settings [3]. The benefits of functional and competent IPC include achieving common goals as the IPC team, which provides mutual benefits for all who are involved in the care of the patient, improves patient outcome efficiency, and decreased costs from litigation cases arising from adverse events [7]. IPC requires an equal share of authority and resources hence, the nature of IPC calls for shared leadership across the interprofessional team [7], [9].

To socialize undergraduate nursing students in interprofessional collaborative practices, during the undergraduate training process, there is allocation in various clinical settings, namely the emergency units, medical wards, and primary healthcare clinics to acquire technical nursing skills, knowledge, and interpersonal attributes that enable them to be safe nursing practitioners [10]. In the allocated clinical settings, the nursing students form part of the interprofessional team and are expected to maintain quality care by providing clear, concise, and accurate communication of their direct patient observation, assessment, and intervention to the rest of the interprofessional team to enable comprehensive care [11]. Whilst functioning within the interprofessional team, the nursing students learn how to demonstrate mutual respect, and recognize authority gradients by being cognisant of the scope of practice of the different health professionals, which is important in decision-making and avoiding power dynamics and conflicts amid the interprofessional team [7]. Therefore, socialization in functioning within an IPC team requires a structured teaching and learning modality to prepare comprehensively, the nursing students for the realities of functioning in an IPC team. Literature reveals that preparing nursing students to be dynamic and competent in an interdisciplinary team requires a multifaceted learning environment that compromises knowledge acquisition, competency in technical skills, and continuous development of professional identity [12], [13], [14]. Currently, problem-based learning (PBL) is a preferred teaching and learning modality in teaching nursing students within higher learning institutions. PBL is regarded as effective in enhancing theory-practice integration, problem-solving, and self-directed learning skills and further promotes critical thinking [11]. In PBL, the nursing students are encouraged to identify their own knowledge, and skills and apply them in solving real-life situations within the context of providing care to patients and communities [11]. Even though the expectation is for the nursing students to function within an interprofessional collaborative function, there is less inclusion of other healthcare professionals in the teaching and learning process of the nursing students.

Interprofessional education (IPE) promotes the advancement of collaborative learning experiences and practices in preparing nursing students for team-based integrated care of patients [7]. The WHO framework for action on the interprofessional education and collaborative practice declares IPE occurs when two or more healthcare professionals learn from and with each other to enable effective and efficient collaboration and improve health outcomes. IPE is therefore a critical approach for preparing nursing students to enter the healthcare profession workforce where teamwork and interprofessional collaboration are important competencies [15]. Despite the abundance of IPE reviews and empirical studies in healthcare profession, there is a paucity of guidance on how IPE contributes to develop competent undergraduate nursing students. This integrative literature review aims summarise the contribution of interprofessional education in nursing education to develop competent undergraduate nurses.

## The Aim

The aim of this manuscript is to summarise the contribution of interprofessional education in nursing education in developing competent undergraduate nursing students.

### Design

The study adopted an integrative literature review as a method of inquiry. Integrative literature is a method that aims to explore existing literature to identify and position the knowledge gap that exists in theory and practice [16]. Furthermore, integrative literature reviews summarise past empirical or theoretical literature to provide a comprehensive understanding of a specific phenomenon [17]. As a benefit, an integrative literature review contributes to theory development and has a direct application to practice [17]. The method was useful to explore existing knowledge, through a review of previous studies regarding interprofessional education in nursing education, to yield a broader understanding on the contribution of IPE in nursing education in developing competent undergraduate nursing students. The results of this integrative literature review have potential to inform curriculum developers, nurse educators and researchers to explore how to integrate IPE in the nursing education.

The study adopted a framework, which includes problem identification, literature search, data analysis, interpretation, and presentation of the results [17]. To search for studies, the authors used the following search items: “Competence,” “Developing,” “Interprofessional education,” “Nursing education” and “Nursing students.” The exploration of the literature was from a global perspective, including all available studies across the globe to ensure a greater understanding of the phenomenon in question. To facilitate the search for literature related to the contribution of interprofessional education in nursing education in developing competent undergraduate nursing students, the review question was as follows:

***“What is the contribution of interprofessional education in nursing education in the developing competent nursing students?”***

### Problem identification

Due to the burden of diseases that predisposes a high number of hospitalised patients, the increase in chronic illness and patients who need complex care, and the significant low number of nursing staff within the healthcare establishments, nursing staff are challenged to be properly equipped with prompt critical thinking, problem solving and decision making that are aligned to the rest of the interdisciplinary team for safe and quality healthcare outcomes [18]. Furthermore, the rapid evolving scientific knowledge necessitates IPC for optimal patient care [19]. The lack of interprofessional collaboration knowledge, skills and attitude within the nursing profession leads to chaotic, unpredictable healthcare establishments, destructive power dynamics, and a strained communication between the interdisciplinary team [18]. This phenomenon poses a great need for co-ordinated interprofessional team work for synergy and quality healthcare provision to the public [18]. In addition, the several healthcare outbreaks, such as COVID-19, have recognised the need for interprofessional collaboration competence of nursing students in managing disaster situations. Despite the above discussion, the current nursing curricular do not include the comprehensive inclusion of IPE in teaching of nursing students. In addition, IPE is effective at student nursing level to develop competent nursing students compared to traditional education, as it enables the knowledge and skills necessary for collaborative teamwork [19].

### Literature search

In the second step of the review process, the authors consulted an experienced librarian from the Health Science faculty to assist in ensuring a credible and in-depth literature search strategy to retrieve the relevant data for the review. There were three databases searched for the articles, namely CINHAL, Scopus, and Science Direct. Articles published between 2018 and 2022 were retrieved using search terms such as “Competence,” “Developing,” “Interprofessional education,” “Nursing Education” and Undergraduate nursing students.” In the context of this review, the review question and the literature search based on the following PIO (Population, Intervention and Outcome) approach. The PIO is adapted from the PICO (Population, Intervention, Comparison and Outcome), which is a widely used strategy to formulate a research question [20]. In the context of this study, there was no comparison, thus there was the adoption of the following PIO approach:

P = Undergraduate nursing students.

I = Interprofessional education.

O = competence.

The search initially resulted in 1350 articles. After excluding the duplicates and studies not related to the PIO approach, there were eight articles included in the review. The inclusion and exclusion criteria adopted for the study are:

### Inclusion criteria

- Published studies empirical which includes Qualitative and Quantitative studies, mixed methods research, reports, reviews relative to contribution of IPE in nursing education in developing competent nursing students.
- Full text studies published between 2018 and 2022.
- Studies published in English.

### Exclusion criteria

- Studies focusing on IPE contribution on non-healthcare allied professions in training.
- Non-English written studies.

**Fig. 1:**
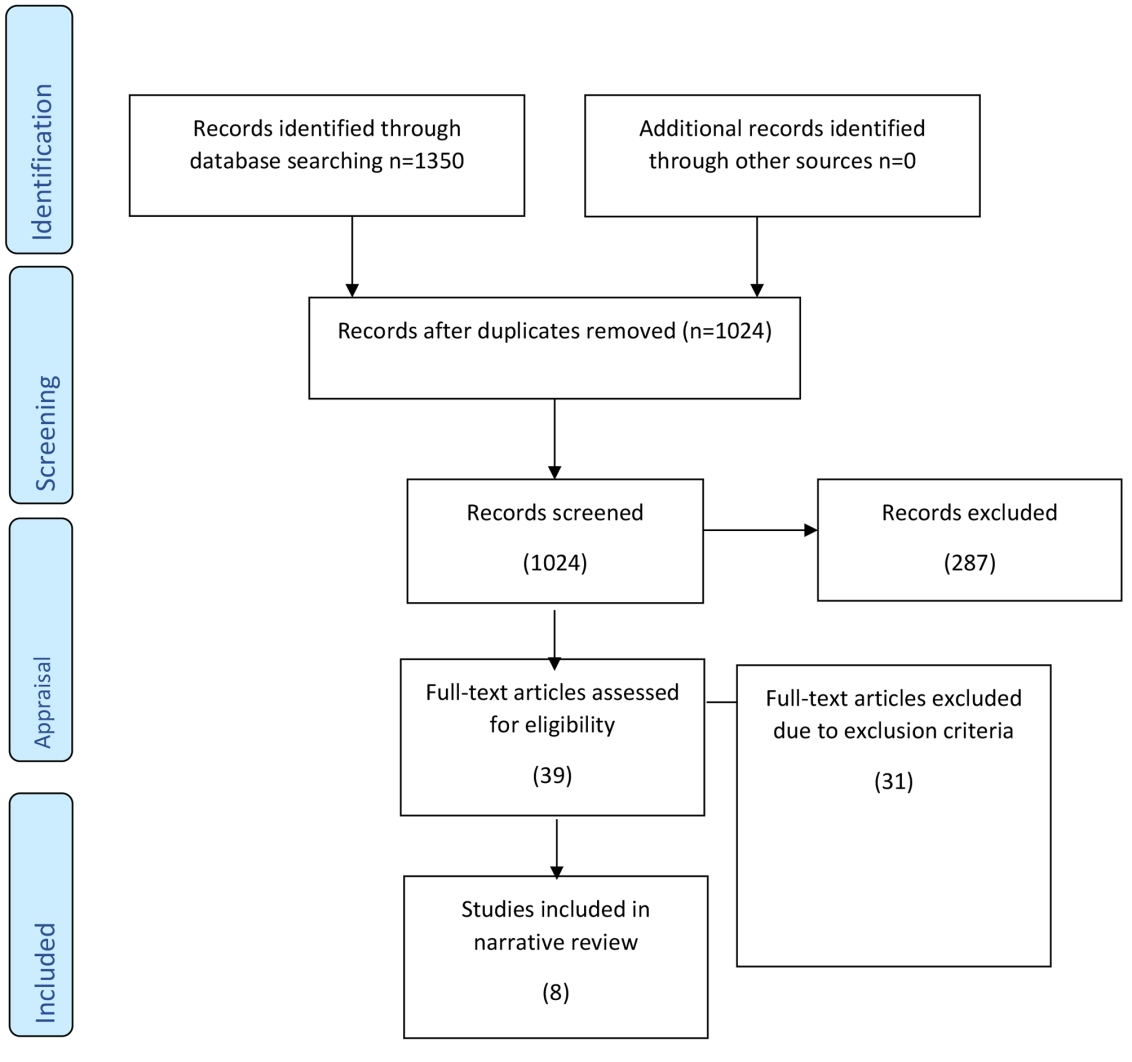
Overview of the search strategy. Source: Adapted from the 2009 PRISMA flow diagram

### Quality appraisal

The included articles in the review underwent critical appraisal using a quality appraisal tool, for studies with diverse designs, known as QATSSD [21]. The tool aimed to measure the quality of articles based on the following four indicators: theoretical framework, aim, sample size and population. The indicators are measured from no mention rated by 0, to explicit mention, rated by 3 [21]; the high value specifies the quality of the indicator that is measured. As the study included a variety of research with different methods, the tool was the best to appraise the research evidence, and the effectiveness [21]. The articles included were independently rated by the first and the second author using the QATSSD. All authors then meet and discussed the discrepancies and reached a final consensus regarding the appraisal rating of the included articles.

### Data analysis

Data analysis requires the data to be orderly, coded, categorised, and summarised into a unified and integrated conclusion about the research problem [17], The unbiased interpretation of the studies along with the synthesis of the evidence are the goals of the data analysis stage. In this stage, the authors of the study analysed the included studies using thematic data analysis. Table 2 presents a summarised findings of the review in the form of three themes and subthemes that emerged after the analysis. In the [17] framework, the data analysis includes data reduction, which involves the determination of the overall classification system for managing the data from diverse methodologies [17]. In this review, the authors divided the sources into subgroups to facilitate the analysis and followed by extracting and coding each included articles to organise the data. A data extraction tool, in the form of a table, is a preferred method to facilitate a standardised extraction of relevant information from the included articles [22]. In this review, the data extraction was in alignment with the purpose and the review question of the review, as suggested by [22] (See Table 1 with the data extraction).

**Table 1:** Data extraction table

Authors/Year	Study Aim		Study Design			Outcome	Sample		Key Findings	
Country						Measures	Characteristics			
Homeyer et al., [1] Mecklenburg-Western Pomerania	To explore, how IPE has to be designed and implemented in medical and nursing training programs to optimise students’ impact for IPC		Qualitative; Delphi method			Expert opinion and content-endorsed categories	25 experts, defined as individuals (a) able to answer every research question, (b) interdisciplinary, (c) sustainable, and (d) appropriate status		The experts found more enablers than barriers for IPE between medical and nursing students	
Gonzalez-Pascual et al., [23]	To describe and analyse the use of a station within an OSCE to assess interprofessional competence performance in undergraduate nursing students		Quantitative; Cross-sectional study			Interprofessional Collaborator Assessment Rubric	Second year nursing students (*n* = 86)		Most students have demonstrated interprofessional competence performance at a good level	
Spain										
Schmidt et al., [24]	To investigate, if the interprofessional team-training of champions can be successfully transferred into clinical practice		Quantitative; pre-post design with one measurement before interprofessional team-trainings, and the second measurement six months after completion of the trainings			German version of the Hospital Survey on Patient Safety Culture	Volunteer Clinicians (*n* = 179)		Participation in the IPE training sessions played a variably relevant role in the communication practices	
Germany										
Goulding et al., [25] Canada	To examine the perceived changes in Bachelor of Science in Nursing (BScN) students and MLSc students’ attitudes toward interprofessional collaboration following a simulation-based interprofessional education (Sim-IPE) activity		Mixed Methods			Simulation Effectiveness Tool–Modified, and Debriefing transcripts	Student volunteers (*n* = 17)		The results demonstrated that students enjoyed learning together and valued interprofessional education	
Wong et al., [18] America	To describe the lived experience of staff members caring for this population to provide a broad perspective of Emergency Department patient violence		Qualitative; phenomenologic approach			Themes identified from focus groups and individual interviews	Volunteer Healthcare Workers (*n* = 31)		Identification of issues that coalesced into four tiers of healthcare delivery at the individual, team, environment, and system levels	
Maranon and Pera [26]	To understand how nursing students at the end of their nursing education view nursing autonomy		Qualitative			Themes identified from focus groups	Third year nursing students (*n* = 23)		The study findings reveal confusion about nursing autonomy in people who will soon be professionals, and they suggest a problem in the definition of the profession	
Spain										
Manspeaker et al., [27]	To expose and teach healthcare students about Interprofessional Education (IPE)		Mixed Methods; Retrospective, pre-test post-test			Readiness for Interprofessional Learning Scale scores, and identified categories	Healthcare students (*n* = 12)		Results of this study reveal that undergraduate students enrolled in various healthcare professions demonstrated change in their overall attitudes toward interprofessional learning, and further appreciation for teamwork, learning, and differing healthcare practices between Australia and the United States following a SA programme	
America										
Spaulding et al., [28]	To assess the impact of IPE on outcomes related to health-care pre-licensure learners and professionals, including changes in attitudes/perceptions; acquisition of knowledge regarding other disciplines’ roles and development of collaborative skills; and change in collaborative behaviour		Systematic review			Narrative	19 published articles		All of the studies assessed modifications in attitudes and perceptions (Barr et al. level 2a), 63% of the studies assessed acquisition of knowledge and skills (Barr et al. level 2b), and 37% of the studies assessed behavioral change. A variety of study designs (e.g., quasi-experimental, mixed methods, and controlled longitudinal) with a comparison group in some studies (Dacey et al., 2010; Darlow et al., 2015; McCaffrey et al., 2013; McFadyen et al., 2010; Pullon et al., 2016), a wide array of settings (e.g., classroom, online, simulation, and clinical), and generally large sample sizes were notable	
America										

### Synthesis

Figure 1 above demonstrates the search strategy adopted in the study. The first and second author, acted as primary coders. Initially the coders organised the data and identified the sources that would be analysed. Individually, the two analysed the data independently. The authors extracted the contributions from the obtained articles and clustered them in the form of themes. The two authors compared the extracted data, rearranging it according to code, and categorised the obtained data. Following a consensus meeting with all the included authors, there was grouping of the categorised data under themes. Three themes emerged from the review, namely promotion of patient safety in nursing practice, socialise nursing students in interprofessional collaboration and promote the development of professional identity. Table 2 provides the emergent main themes and the subthemes.

## Results

Three themes emerged from the findings of this study, namely promotion of patient safety in nursing practice, socialising the nursing students in interprofessional collaboration, and promoting the development of professional identity. Table 2 presents the themes and sub-themes.

**Table 2:** Themes with subthemes

Main Theme	Subtheme
1. Promotion of patient safety in nursing practice	1.1 Patient safety culture 1.2 Training on teamwork strategies 1.3 Patient-centred care
2. Socialising the nursing students in interprofessional collaboration	2.1 Role clarification 2.2 Effective team communication & Effect teamwork abilities
3. Promoting the development of professional identity	3.1 Improved self-awareness 3.2 Development of Interprofessional identity

### Theme 1: promote patient safety in nursing practice

Promotion of patient safety in nursing practice emerged as the first theme in this integrative literature review. Sub-themes include patient safety culture, training on teamwork strategies and patient-centred care.

### Patient safety culture

Patient safety is regarded a global issue, due to medical errors being the leading cause of death across the globe [29]. A study estimated that between 44 000 and 98 000 patients die a year because of medical errors [23]. Medical errors cannot be blamed solely on one specific profession, as there are multiple factors that contribute to the medical error occurrences. Medical errors are much more complex in healthcare systems such including in mental health care settings, where there is a vast interaction of multiple professions as well as technology use; these medical errors can compromise the quality of patient care and safety [29]. To mitigate the factors that lead to medical errors, it is necessary to establish and maintain a culture of patient safety, with the involvement of all the healthcare professionals providing care to the patients and communities. Furthermore, literature reveals that patient safety culture is identified as a core factor for improving patient safety in healthcare organisations. The term is defined as the product of not only individuals but group values, attitudes, competencies, and patterns of behaviour that regulate the commitment proficiency of an organisation’s health and safety management. Patient safety culture has been examined to hold great effects on patient safety outcomes globally [30, 31]. Additionally, the vital component of patient safety culture includes a mutual belief that the risk of responsibility is high, organisational commitment to identify and analyse errors from the patients and ultimately creating an environment that balances the need of medical errors reporting and the need for disciplinary, thus patient culture reinforces to healthcare teams to prioritise patient safety in the manner and scope they provide patient care [32]. A cross-sectional study, conducted in both the public and private hospitals in emergency wards within Tunesia showed that patient safety culture in the highly interprofessional team setting is a great need it reinforces teamwork, where the healthcare professionals are transparent with their work, adopts effective communication to provide comprehensive care to the patients. Furthermore, the study has shown, that although the setup in the emergency wards is complex and at times impossible to keep patients informed [33]. Patient safety culture reinforces openness where the is feedback on treatment and honesty in prognosis to release anxiety in patients. Moreover, it has been shown to limit the occurrence of adverse events and a non-punitive response to errors in a potentially hazardous environment [33].

In the review, [23] suggested creating a culture of patient safety, where undergraduate nursing students have to work intensively with interprofessional teams and learn how to communicate effectively among the team and understand the team’s individual roles and functioning. The understanding of team function and role clarification will position the nursing students to make clinical decisions that are in alignment with the rest of the IPE team, and further limit team dynamics and conflicts that can create animosity within the interprofessional collaboration team [23]. The same authors further allude that IPE, with interprofessional collaboration from an undergraduate level, will deepen the team functioning of nursing students through building trust, respect, judgment, and capabilities, as well as being sensitive to the IPE collaboration team safety concern [23]. Thus, IPE education will promote the acquisition of teamwork skills, which is an essential skill in endorsing patient safety [23].

In affirmation to the views of the authors above, stated that IPE is a key component in reducing preventable medical errors through the promotion of communication, role clarification, and mutual respect among nursing students and other healthcare professionals [25]. Further, with the adoption of interprofessional education, using teaching strategies such as simulation-based interprofessional education (Sim-PE), nursing students at the undergraduate level will be able to enhance how they perform clinical skills as well as develop their critical thinking skills whilst working in a safe and collaborative setting [25]. The authors provided examples such as use of virtual reality, role-playing, full body mannikins, including other healthcare professional training students to mirror realistic scenarios for collaborative training and as a form of reflection to enhance their clinical reasoning and problem-solving skills.

### Training on team-work strategies

The second subtheme that emerged was training of teamwork strategies, for efficient diagnostic, team-based treatment, and care at undergraduate nursing education level. The team-based teaching approach imposes patient safety standards to nursing students and other healthcare professionals within the clinical settings [24]. The authors further recommended that to improve efficiency, quality, and patient safety, the Team Strategies and Tools to Enhance Performance and Patient Safety (TeamSTEPPS), as a training approach, would provide key interprofessional competencies. IPE competencies involve taking accountability or effective leadership, understanding into how to effectively close the loop in communication, how to effectively monitor clinical situation, how to provide mutual support in an IPC team, how to be team orientated and share same mental model with the rest of the IPC team [24]. The training includes skills on, Background, Assessment and Recommendation (SBAR), a technique recommended for prompt and appropriate communication within healthcare settings, especially for nurses with other healthcare professions, such as physicians [24].

Furthermore, since complex care involves an interprofessional team, which has different professional backgrounds, this requires frequent handover and transitions; therefore, interprofessional team communication through IPE is crucial in ensuring there is effective information transmitted along the patient care process [24]. The studies in the review revealed that techniques such as two-way communication, feedback, and briefings could support nursing students and other healthcare professionals’ communication, hence contributing to improved quality and safety of care [24]; [25].

### Patient-centered care

The third subtheme was IPE promoting nursing students to provide patient-centered care. According to [34], IPE in nursing education led to stronger patient-centered care, given the sharing of knowledge that occurs across disciplines. A qualitative study by [1] in Germany, explored the effects of IPE in nursing students and affirmed that IPE on a patient level improves patient-centered care. The IPE enables the nursing students and the rest of the interprofessional team to work well together to coordinate more individualized care, as well as collaboratively plan and design a collaborative treatment suited for the identified holistic needs of the patients. In addition, [1] maintained that the collaborative approach improves efficiency and the quality of patient care, as well as patient satisfaction due to the comprehensive patient-centered approach provided.

### Theme 2: socialise nursing students in interprofessional collaboration

Socialising nursing students in interprofessional collaboration emerged as the second theme in this review. Sub-themes included effective team communication, role clarification and effective teamwork abilities.

### Effective team communication & effective teamwork abilities

The review revealed that IPE socialises nursing students, at an undergraduate level, to work effectively in an interprofessional collaboration. According to [35], it is important for nursing students and the rest of the multidisciplinary healthcare professionals to respect the unique values, roles, responsibilities, cultures, and expertise of other health professionals. Furthermore, the understanding of working in an interprofessional collaboration makes the nursing students maintain confidentiality in the delivery of team-based care and enables them to understand how to communicate effectively across diverse cultures [35]. In addition, [36] concurred with the study and stated that it is feasible that nursing students have good communication and teamwork skills, the skills are a significant positive factor of IPE outcomes, especially in simulated interprofessional education settings, where nursing students learn with other healthcare professionals in a controlled clinical environment.

According to [36] further mentioned that IPE provides nursing students with experiential learning opportunities focused on managing real life clinical situations through communication, teamwork, and collaborated problem-solving which is essential. This ensures that nursing students are equipped and ready to practice in an interprofessional team after undergraduate training.

In addition, [37] highlighted that IPE hinders stereotypes, group favouritism, and out-of-group discriminatory bias among nursing students and other healthcare professionals, which apparently cause animosity and a negative working environment for healthcare professionals in clinical settings.

A study by [37] revealed IPE exposes nursing students to roles they did not know exist within the clinical settings, such as discharge coordinators, and in the process were able to identify how to work better in a team. Through IPE, nursing students become aware of the contribution of the other healthcare professionals within the larger context of the healthcare team, and the way each profession contributes to the patients’ experience whilst in the clinical settings. The knowledge of other healthcare professionals encourages positive consultation with the interprofessional team, thus improving patient care and addressing transition of care concerns using a team-based approach [37]. In addition, [38] indicated that most of the interprofessional dilemmas are due to healthcare professionals’ lack of awareness of their extra-disciplinary colleagues’ roles, experiences, and expertise. IPE in nursing education equips nursing students to identify and expand their perspectives beyond their own professional roles to a more shared understanding of all professional roles for collaboration with limited interprofessional dilemmas. Thus, nursing students are able to manage and handle conflict within an IPC team effectively.

### Role clarification

The review indicated how IPE assist nursing students to clarify roles and functions of other healthcare professionals to better position themselves in the interprofessional collaboration team. In a study by [27], the nursing students and other healthcare professionals involved in a guided discussion in an IPE training programme explained that IPE allowed them to gain a better understanding of how other professions approach patient care. In understanding the phenomenon, the nursing students felt they could better support the team for the priority of ensuring quality patient care. Moreover, IPE in the perceptive of the nursing students revealed a positive learning experience and increase the nursing student’s cultural competence and teamwork [27].

### Theme 3: promote the development of professional identity

Promote the development of professional identity emerged as the third theme in this review. Sub-themes included promotion of self-awareness and development of interprofessional identity. According to [26], professional identity occurs when an individual, in relation to the profession, associates with demonstrate norms, values, behaviour, attitude, and culture required for optimal functioning in the profession. In nursing, professional identity is acquired when the nursing students receive their basic nursing education, receive theoretical knowledge in the classroom, and interact with patients, experienced nurses, and the multidisciplinary team in practice [26].

### Promotion of self-awareness

In this theme, one of the emergent subthemes was the promotion of self-awareness. A study by [39] revealed that IPE aided nursing students to see new options for clinical situations, being able to speak up respectfully for the patients and themselves and further value their practice and role in the interprofessional team, and helped them to understand their own limitations and facilitate a shared learning experience with other healthcare professional students and professionals. The same authors stated that self-awareness plays a crucial role in the professional identity development process of nursing students because nurses can better understand themselves as an individual profession, and fully experience themselves as separate and unique professionals. In the self-awareness process, nursing students are able to empower themselves, create necessary changes in their professional identity and build on areas of strength. Moreover, nursing students are able to identify areas they need to improve on, prompted by the deeper comprehension, change in perspective, and realization of how learning experience is applied to a real-world situation within a team-based approach [39].

### Development of interprofessional identity

The second subtheme that emerged was development of interprofessional identity. [40] define interprofessional identity as an individual’s identification within a wider interprofessional group. In nursing students, IPE enables them to broaden existing ideas of professional identity beyond the nursing profession. According to [40] health professionals who lack interprofessional identity resist interprofessional collaboration to maintain their own professional identity. The disciplinary isolation limits both abilities to understand other professional roles and opportunities to train and be efficient as part of an interprofessional team.

## Discussion

Studies have shown that poor interprofessional team collaboration is key in contributing to avoidable medical errors in the clinical settings [41], [15], [42]. Nursing students are at the forefront of either committing medical errors or being affected by medical errors due to working directly with the patients, high technological equipment and the interprofessional teams [43]. This encourages nursing education to invest in embedding a patient safety culture and practice in nursing students to avoid medical errors [39]. This review has revealed that IPE in nursing education plays a crucial element in developing competent nursing students during the early phases of the nursing students training.

The study has revealed that IPE reinforces effective interprofessional collaboration competencies in nursing students, which facilitates them to provide safe patient carefree from adverse events that could affect the well-being of the patients and communities [39]. The interprofessional competencies include effective communication skills with the interprofessional team, managing team dynamics, being able to clarify the roles, and understanding the impact each has on the contribution of the patients’ experience whilst in the clinical settings. The review revealed that interprofessional competencies reinforce patient-centered care instead of task-orientated care. Patient-centered care is believed to be linked with practices that improve a variety of health conditions, increase patient satisfaction rates, increase adherence to treatment, and improve the quality of care the patient receives [44].

Furthermore, the review has revealed that IPE in nursing education equips nursing students to better assess and diagnose the patient using a team-based approach, facilitated using the IPE teaching approach. Studies reveal that just like nurses, nursing students, when in the clinical settings for workplace integration, play a leading role in communicating valuable information to the interprofessional team, which sustains patient safety [45], [43] should nursing students demonstrate poor communication and poor teamwork, medical errors, such as delayed or missed nursing care, can occur. Implementing the Team Strategies and Tools to Enhance Performance and Patient Safety (TeamsSTEEPS) within the nursing curriculum can improve communication and therefore create competent nursing students who can create and sustain safe patient environments [45].

The review has also highlighted that IPE contributes significantly to socialising nursing students to be able to work effectively within an interprofessional collaboration team. Thus, to build a collaborative clinical setting the IPE is an inherent prerequisite for interprofessional education in nursing education to provide intentional skill acquisition and insight into interprofessional collaborative practice [28]. The review further discussed that ideally, the viewing of IPE should be as a continuum, and commence early during pre-licensure of nursing students and further extend throughout the nursing students’ professional career. Early socialisation to interprofessional collaboration practice enables nursing students to identify the different roles, cultures, and expertise of the interprofessional team, and ultimately assists in enabling them to effectively communicate across diverse cultures without being culturally insensitive or crossing boundaries that can lead to workplace rivalries [28].

In addition, the review revealed that IPE in nursing education assists in developing the professional identity of the nursing students. They become more self-aware about their role and function within a team, build their assertiveness to be able to stand up for themselves, and advocate effectively for the benefit of the patients and communities in their care. The developed professional identity through IPE enhances the nursing students’ confidence in their expected professional role, which enables them to become competent professionals when in IPC team [40]. In this era of evolving healthcare expectations in nursing, the contribution of IPE in nursing students’ enables change agents and aspiring nurses who will have an open mindset to the effectiveness of teamwork in clinical practice.

### Limitations

IPE has been acknowledged since 1988 by the World Federation of Medical Education, and the recommendation was for doctors and other healthcare-allied professions globally to provide training in association with other health professions; the recommendation was later reinforced in 1994 and by the WHO in 2010 [46]. The review only included studies from 2018 to 2022 and missed capturing the perception of IPE education contribution in its conceptualization in nursing education for a comprehensive report. In addition, the study only searched for articles in three databases, which limited the number of the included studies in the review. In the included studies, there were limited studies conducted in an African context regarding the contribution of IPE in nursing education, which meant the authors were not able to distinguish or compare IPE contributions in nursing education between the developed and developing countries.

### Recommendations

There can be practical strategies adopted to integrate IPE within the nursing education curriculum, such as peer teaching across medical and social science departments, especially when learning concepts critically linked to nursing and other healthcare-allied students, such as ethics and professionalism. The higher learning institutions, in collaboration with the clinical facilities, can develop quality improvement projects, where the nursing students and other healthcare allied students can work with each other to improve service gaps identified within the clinical facilities and the higher learning institutions; in this way, the nursing students become aware of the scope of other healthcare professions in the facilities. The implementation of the collaborative quality improvement projects can help to reinforce IPE collaboration competence and enhance IPE competencies amongst the nursing students. Furthermore, the nursing schools can develop scholarly teaching and learning proposals with other healthcare allied schools within the health sciences that promote IPE in the teaching and learning of nurses. Therefore, the innovative IPE teaching strategies can broaden the professional identity of the nursing students to avoid professional silos, and readily prepare the nursing students for real-life clinical situations post-licensure training in the process of developing an interprofessional identity, as recommended by the review.

In terms of research, to broaden the knowledge on the phenomenon of this review, further studies, which can either be Qualitative, Quantitative or Mixed method, can be conducted to explore the readiness of African nursing students in IPE, and explore best practice strategies that can be adopted in the nursing curriculum to reinforce IPE collaboration, especially within the African countries, as there were limited studies retrieved in that respect. In addition, qualitative/quantitative or mixed method studies can further explore the perceptions of nursing curriculum developers, nursing educators in IPE collaboration and education to measure the readiness and effectiveness of IPE education from their perspective. The results from the indicated studies can inform the nursing regulatory bodies to consider including IPE education within the nursing curriculum to benefit fully from the contributions of IPE in nursing education.

## Conclusions

This study serves as a foundation for exploring the incorporation of interprofessional education approach in nursing students training during undergraduate training level. The nursing students, through IPE, are equipped to be more professional in their communication, and provide safe and quality patient care. Furthermore, IPE assists nursing students to better position themselves in an interprofessional team without being timid or engulfed with fear and gives them a more interprofessional identity, where the nursing students identify themselves as part of dual professionalism with other healthcare professionals. Nursing students become more confident and competent in their role with the effects of IPE.

## Data Availability

The datasets used and/or analyzed during the current study are available from the corresponding author upon reasonable request.

## Declarations

### Disclaimer

The views and opinions expressed in this manuscript are those of an author, and do not intend to communicate the stance of the institution or affiliation of the author.
